# Creation of 3D Multi-Body Orthodontic Models by Using Independent Imaging Sensors

**DOI:** 10.3390/s130202033

**Published:** 2013-02-05

**Authors:** Sandro Barone, Alessandro Paoli, Armando Viviano Razionale

**Affiliations:** Department of Civil and Industrial Engineering, University of Pisa, Largo Lucio Lazzarino, n.1–56126 Pisa, Italy; E-Mails: s.barone@ing.unipi.it (S.B.); a.razionale@ing.unipi.it (A.V.R.)

**Keywords:** dental CBCT images, optical scanning, sensor fusion, tooth segmentation, orthodontic model

## Abstract

In the field of dental health care, plaster models combined with 2D radiographs are widely used in clinical practice for orthodontic diagnoses. However, complex malocclusions can be better analyzed by exploiting 3D digital dental models, which allow virtual simulations and treatment planning processes. In this paper, dental data captured by independent imaging sensors are fused to create multi-body orthodontic models composed of teeth, oral soft tissues and alveolar bone structures. The methodology is based on integrating Cone-Beam Computed Tomography (CBCT) and surface structured light scanning. The optical scanner is used to reconstruct tooth crowns and soft tissues (visible surfaces) through the digitalization of both patients' mouth impressions and plaster casts. These data are also used to guide the segmentation of internal dental tissues by processing CBCT data sets. The 3D individual dental tissues obtained by the optical scanner and the CBCT sensor are fused within multi-body orthodontic models without human supervisions to identify target anatomical structures. The final multi-body models represent valuable virtual platforms to clinical diagnostic and treatment planning.

## 1. Introduction

The development of procedures for the computerized design and manufacturing of custom dental devices has become of growing interest to orthognathic and orthodontic treatments. In clinical practice, odontoiatric diagnosis and therapy planning conventionally rely on the use of plaster models of the patient's mouth. Moreover, two-dimensional lateral cephalograms and/or panoramic radiographs are often used to assess clinical outcomes and to provide information about the relative disposition between dentition and skeletal structures. However, the use of 2D investigations does not always provide reliable information about relative displacements between teeth, roots and bone. In case of complex pathologies (*i.e.*, unerupted or impacted teeth, presence of severely curved roots), full 3D digital dental models should be used to predict the biomechanical behavior of dental tissues and to evaluate the biological feasibility of treatment plans. Generally, computer-assisted dentistry requires a digital virtual model providing the following features: (i) accurate modeling of tooth crown surface and related soft tissue (gingiva), (ii) reconstruction of alveolar bone structure and tooth root geometry, and (iii) reliable relative placements of dental tissues. Within restorative dentistry, orthodontics requires the reconstruction of a full mouth model (orthodontic model) to provide better functionalities and appearances. In this context, the gingiva model is essential to control the motion of teeth within visually acceptable conditions. Moreover, root geometry is required to analyze pathways of tooth movements during the treatment over time, especially for complex malocclusions [1].

Nowadays, orthodontic clinicians can be assisted in malocclusion diagnoses and virtual treatment planning by 3D imaging techniques such as computed tomography (CT), magnetic resonance (MR), stereo-photogrammetry and optical scanning. However, none of the existing imaging technologies are able to simultaneously acquire and integrate all the anatomical tissues that are involved in the clinical orthodontic practice.

Computed Tomography is considered the first choice for demanding bone imaging tasks, even if high radiation doses are unavoidable. In recent years, Cone Beam Computed Tomography (CBCT) has been introduced in dentistry and orthodontic applications since diagnostics accuracies are obtained with lower radiation doses [2]. However, CBCT data do not provide images suitable for accurate 3D reconstructions of soft tissues. The presence of artifacts owing to metal restorations and/or orthodontic fixed appliances, impairs the accurate reproduction of tooth information. Moreover, accuracy and resolution of CBCT reconstructions are not adequate for the design and production of tight-fitting removable appliances. On the other hand, optical scanning can be effectively used to provide accurate digitalization of patient's dental arches, also reproducing oral soft tissues. However, surface optical scanners only provide the reconstruction of visible surfaces, whereas bone structures and teeth roots are missing.

In recent years, complete models of dental structures are typically obtained through the fusion of multi-modal data obtained by integrating different imaging sensors. Technical literature has documented the use of multi-modal image fusion processes for the creation of facial skeleton–dentition models by integrating digital patient's teeth captured by an optical scanner within bone models reconstructed by tomographic scanning. These approaches establish an augmentation of skeletal models with improved visualization of dentition without artifacts [3–5]. However, none of the proposed solutions takes into account the reconstruction of individual tooth shapes including root morphology. In [6], a method for visualizing tooth roots within orthodontic models has been experienced by integrating information from CBCT and optical surface scanning. However, 3D CBCT tooth models including the roots were manually segmented and reconstructed by using commercial software though outlining and masking tools which required labor intensive and time consuming efforts.

In this paper, a 3D data fusion methodology has been developed in order to create reliable multi-body orthodontic models by using a Cone-Beam Computed Tomography (CBCT) device and a structured light scanning technique. The optical scanner is used to create an accurate digital impression model composed of visible dentition structures (tooth crowns) and oral soft tissues, through the digitalization of both patient's mouth impression and the respective plaster model. The digital impression model is then used to perform a 3D surface segmentation of tooth crowns on the basis of a local estimate of the curvature information. Moreover, the optically-scanned crown models are used to guide the segmentation of CBCT images. In particular, tooth crowns and roots are individually reconstructed by processing CBCT data sets on the basis of an active contour model in a level set formulation.

The final orthodontic model is provided by the fusion of the multi-modal data sets including the most accurate representation for each tissue: *i.e.*, tooth crowns and gingiva by optical scanning and tooth roots and alveolar bone by CBCT imaging. The creation of multi-body dental models allows clinicians to simulate and visualize orthodontic treatments. In the paper, the reconstruction of a full 3D digital model, relative to a complex case with impacted teeth, is finally presented and discussed.

## 2. Methods

The proposed methodology exploits 3D digital tooth crown models obtained by an optical scanning technique. In particular, the optical scanner is used to digitize plaster models manufactured from mouth impressions, which still represents the most accurate replicas of patients' dentitions [7]. The tooth crown reconstruction is further enhanced by also scanning the mouth impression models. The two distinct scanning results are merged and used to separate each individual crown surface from the oral soft tissue geometry by exploiting the local curvature map. This approach allows a better modeling of the boundaries between adjacent teeth, which typically present missing data at touching regions.

The CBCT image processing provides roots and jaws. In particular, tooth crown geometries obtained by the optical scanner are used to guide the reconstruction of root morphology and alveolar bone by optimizing the detection of tooth-bone boundaries, which are hardly distinguishable.

**Figure 1.**
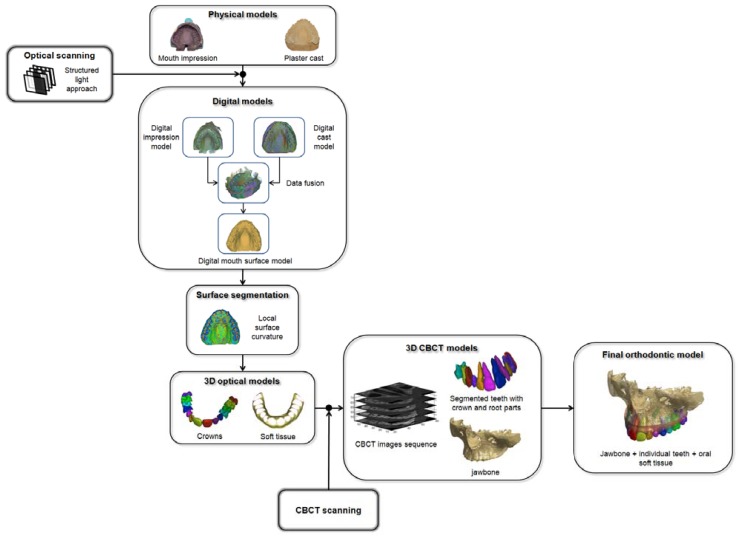
Scheme of the overall methodology to create reliable orthodontic models.

The final step consists in merging the multi-modal dental data in order to provide an accurate full orthodontic representation including individual crowns obtained by the optical scanner and roots reconstructed by the CBCT. Moreover, the overall procedure provides each tooth in a reliable placement with respect to the alveolar bone. The overall methodology is schematized by the workflow shown in Figure 1 and described in the following sections.

### 2.1. Digital Mouth Model through Optical Scanning

**Figure 2.**
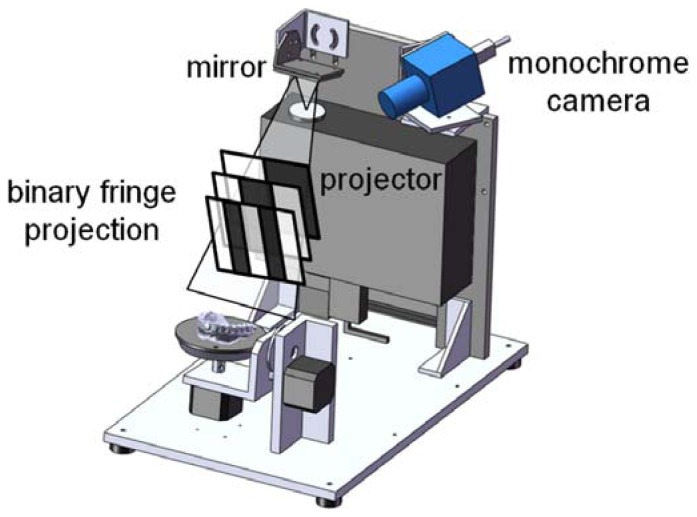
Scheme of the assembled optical dental scanner.

In this paper, an optical scanner based on an active stereo vision approach (Figure 2) has been assembled in order to reconstruct patients' dentition models including tooth crowns and surrounding gingival tissue [8]. Typically, these models can be either obtained by scanning the inner surface of an impression or the outer surface of a plaster cast. However, not all the surfaces composing a tooth shape can be easily reconstructed by using an optical scanning methodology. In particular, two circumstances may occur: (1) the space between the proximal surfaces of adjoining teeth (interproximal space) is not accessible to the impression material and therefore cannot be captured; (2) the interproximal space is adequate to be captured by the impression, but not sufficient to avoid optical undercuts during the plaster model scanning. In the first case, crowns remain incomplete either by scanning the impression or the plaster cast since geometry details of interproximal regions, where adjacent teeth in the same arch are contacting, are missing. The interproximal space must then be reconstructed by post-processing the acquired data through hole filling tools which, however, only approximate the real surface geometry by interpolating neighborhood data. In the latter case, the interproximal space can be reconstructed by scanning the inner surface of the patient's impression.

In this paper, the optical scanner has been configured with the aim at digitizing both impressions and plaster casts through the integration of an optical head with a motorized platform. A two-axis platform structure, including rotating and tilting movements, has been designed by assembling a turntable equipped with two stepper motors having a resolution of 400 steps per round.

The optical sensor (Figure 2) is composed of a monochrome digital CCD camera (1,280 × 960 pixels) and a multimedia white light DLP projector (1,024 × 768 pixels) which are used as active devices for a stereo triangulation process. In this paper, a multi-temporal Gray Code Phase Shift Profilometry (GCPSP) method is used for the 3D shape recovery. A sequence of vertical light planes is projected onto the model to be reconstructed. The planes are defined by black and white fringes whose period is progressively halved along the temporal sequence. Each pixel in the camera images is characterized by a light intensity that can be either bright or dark, depending on its location in the respective plane image. A binary code (0, 1 with n bit) is assigned to each pixel, where *n* is the number of the projected stripe patterns, and the values 0 and 1 are associated to the intensity levels, *i.e.*, 0 = black and 1 = white. This encoding procedure provides l = 2^n^−1 encoded lines. The 3-D coordinates of the observed scene point are then computed by intersecting the optical ray with the plane considering that the geometry of the hardware set-up, the camera ray direction and the plane equation of the corresponding stripe are known. The methodology provides *n_p_* = *l_h_* × *l_v_* encoded points, where *l_h_* is the horizontal resolution of the projector while *l_v_* is the vertical resolution of the camera.

The optical reconstructions are carried out by collecting 3D surface data of dental models from various conveniently selected directions. Different views are automatically aligned with reference to a common coordinate system on the basis of accurate angle measurements around the controlled rotating axes, exploiting a calibration procedure which relates the turntable position with respect to the common reference system [3]. The combination of two distinct controlled axes allows a reliable reproduction of shape details, since different viewing directions better handle occlusion problems and undercut areas.

The vision system has been configured for a working distance of 300 mm and a working volume of 100 mm × 80 mm × 80 mm (width × height × depth). The scanner is capable of measuring about 1 million 3D points with a spatial resolution of 0.1 mm and an overall accuracy of 0.01 mm [3]. The integration between optical devices and mechanical turntables allows the automatic full field data capturing and reconstruction of dental casts and patient's impressions.

**Figure 3.**
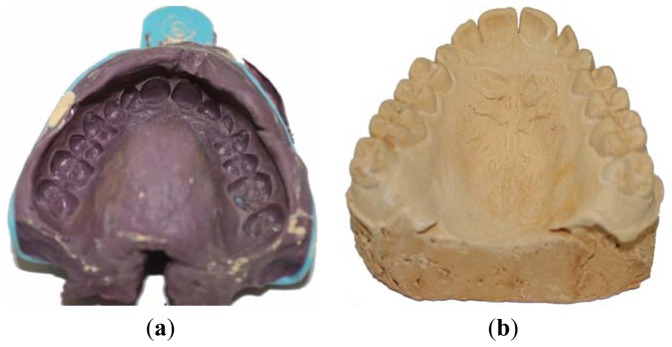
(**a**) Patient's mouth impression. (**b**) Corresponding plaster cast.

**Figure 4.**
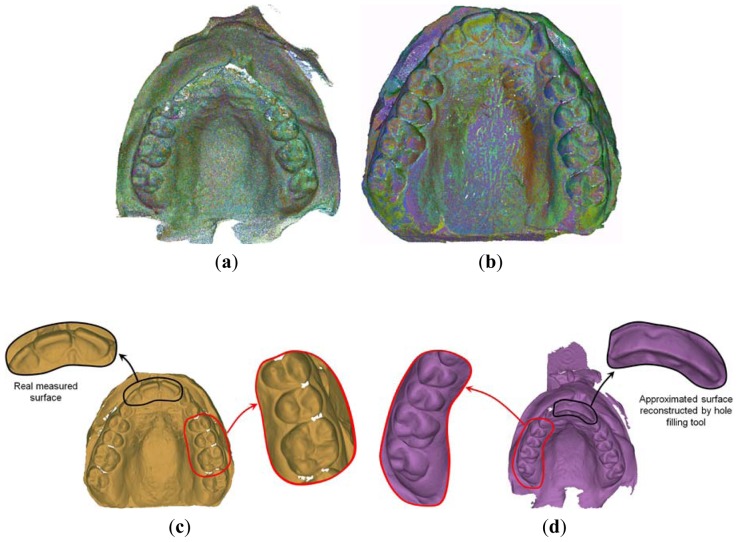
Aligned scans relative to the acquisition of the mouth impression (**a**) and the plaster cast (**b**) along with the corresponding triangular mesh models (**c**,**d**).

Figure 3(a,b) show an example of patient's mouth impression (made by polyether impression material) along with the corresponding manufactured plaster cast (made by yellow stone), respectively. Figure 4(a,b) report the 3D reconstructions of the two physical models as obtained by aligning 16 different views for each model. As clearly visible in the corresponding triangular mesh models (Figure 4(c,d)), interproximal spaces are better reconstructed by scanning inner surface of the patient's impression, whereas the top of the teeth is better acquired by scanning the outer surface of the plaster cast.

**Figure 5.**
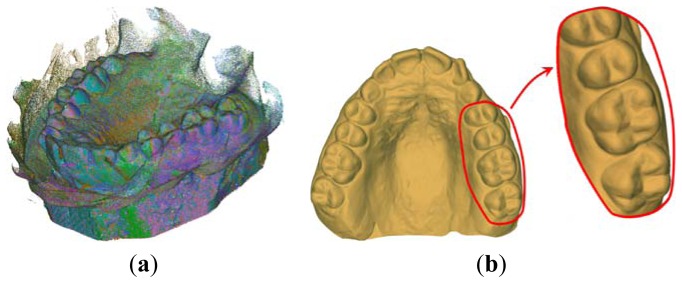
(**a**) Fusion of the different views of the two distinct data sets. (**b**) Corresponding digital mouth surface model.

Figure 5 shows the final digital reproduction of the patient tooth crowns and surrounding gingival tissue (digital mouth model) as obtained by fusing the captured data. The two distinct data sets are integrated through a manual alignment which is refined by a global registration procedure based on ICP techniques (Figure 5(a)). The final result is obtained by merging the common areas between the impression and the plaster digital models within a predefined tolerance value. A classical reconstruction pipeline (filtering, sampling and marching cube tessellation) [9] is then used to obtain the StL digital representation of the mouth surface model (Figure 5(b)).

### 2.2. Segmentation of Tooth Crown Surfaces

The overall surface representing tooth crowns and oral soft tissue must be segmented into disconnected regions representing the individual tooth shapes and the gingiva. The mesh model can be partitioned by computer-based cutting tools, even if a manual process would require labor-intensive interactions. In this paper, a semi-automated procedure has been developed by exploiting the curvature of the digital mouth model. This model contains ridges and margin lines, which highlight the boundaries between different teeth, and between teeth and soft tissue. Regions with abrupt shape variations can be outlined by using curvature information.

In this paper, curvature estimations are efficiently computed by directly processing the acquired sample points without exploiting any intermediate tessellation. In particular, local surface properties can be computed on the basis of the eigenanalysis of the covariance matrix of local neighborhoods of sample points [10]. The covariance matrix for a sample point ***p*** is given by:

(1) $${\text{C}}_{p}={\left[\begin{array}{c} {\text{p}}_{i_{1}}-\bar{\text{p}}\\ \cdots \\ {\text{p}}_{i_{k}}-\bar{\text{p}} \end{array}\right]}^{T}\left[\begin{array}{c} {\text{p}}_{i_{1}}-\bar{\text{p}}\\ \cdots \\ {\text{p}}_{i_{k}}-\bar{\text{p}} \end{array}\right]i_{j}\in N_{p}$$

and:

(2) $$\bar{\text{p}}=\frac{1}{N_{p}}\cdot \sum_{i_{j}=1}^{N_{p}}{\text{p}}_{i_{j}}$$

where ***p̄*** is the centroid of the neighbors ***p**_i_j__* of ***p*** and *N_p_* is the number of points within the neighborhood. Since ***C*** is symmetric and positive semi-definite, all eigenvalues *λ_l_* (*l*∈{0,1,2}; are real-valued and the eigenvectors ***v*** *_l_* form an orthogonal frame, corresponding to the principal components of the point set *N_p_*. Eigenvalues *λ_l_* measure the coordinates variation of points *p_i_* along the direction of the corresponding eigenvector. Assuming *λ_0_* ≤ *λ_1_* ≤ *λ_2_*, the plane:

(3) $$T(\text{x}):(\text{x}-\bar{\text{p}})\cdot {\text{v}}_{0}=0$$

through ***p̄*** minimizes the sum of squared distances to the neighbors of ***p***. Thus, the eigenvector ***v*** *_0_* approximates the surface normal ***n****_p_* at point ***p***, whereas ***v****_1_* and ***v****_2_* describe the tangent plane at point ***p***. The eigenvalue *λ_0_* quantitatively describes the points variation along the surface normal, thus providing an estimate of the points deviation from the tangent plane. A curvature parameter *δ_n_*(***p***), at each point ***p*** in a neighborhood of size *n*, can then be defined as [10]:

(4) $$\delta_{n}(\text{p})=\frac{\lambda_{0}}{\lambda_{0}+\lambda_{1}+\lambda_{2}}$$

If *δ_n_*(***p***) = 0, then all the neighboring points of ***p*** perfectly lie on the plane, if *δ_n_*(***p***) = 1/3 then a perfect isotropic points distribution can be assumed.

**Figure 6.**
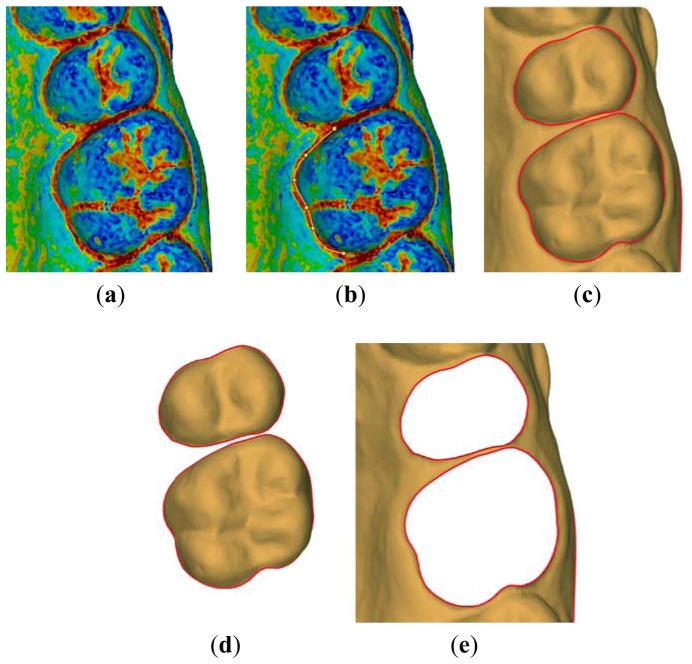
(**a**) Map of the surface curvature distribution. (**b**) Selection of interpolation points. (**c**) cubic splines reproducing the margin line. Segmentation of the tooth crown (**d**) and gingiva (**e**).

The curvature parameter is clearly influenced by the noise occurring in the acquired points, the point sampling density and the neighborhood size used for the computation process. Therefore, the neighborhood size should be estimated by empirical analyses [11]. In this paper, a spherical neighborhood with radius 2 mm has been used. Figure 6(a) shows a detail of the surface curvature map around two teeth.

**Figure 7.**
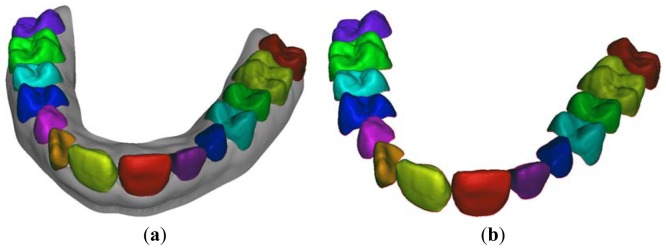
(**a**) Segmented digital merged model and (**b**) relative optically-scanned crown model.

The curvature information is then used to separate crown surfaces from gingival tissue (Figure 6(d,e)) by using cubic splines which are designed by interpolating few points manually selected around each individual tooth (Figure 6(b,c)). Figure 7 shows the result of the crown surface segmentation displaying individual teeth along with the gingival tissue by using different colors.

### 2.3. Segmentation of CBCT Volumes

One of the most challenging issues for 3D dental modeling from CBCT images concerns with the segmentation and manipulation of individual teeth. Several factors may influence the tooth detection process: (i) images deriving from CBCT can be noisy due to low radiation doses, (ii) tooth crowns may have touching adjacent regions occurring in some slices, (iii) dental fillings and/or orthodontic fixed appliances usually cause streak artifacts that may degrade the target data. All these circumstances impair the accurate extraction of individual tooth shapes.

In this paper, a multi-step tooth segmentation procedure has been developed by integrating a thresholding method and a level set method. In particular, the overall procedure is guided by the result of the 3D tooth surface segmentation performed onto the digital mouth surface model (Figure 7(b)). The aim of the proposed approach is twofold. Firstly, a reliable placement of the crown shapes, captured by the optical scanner, is obtained with respect to the bone structure reconstructed from CBCT imaging. Secondly, accurate tooth root geometries are extracted with minimal human intervention.

The methodology consists in the following steps:

- 3D crown reconstruction by segmenting CBCT images through different threshold values;
- alignment of the optically-scanned crown model (ground truth) to the segmented CBCT crown volumes by determining the optimal threshold value which minimizes the alignment discrepancies;
- individual reconstruction of tooth roots guided by the crown shapes extracted in the segmentation step performed onto the surface mouth model;
- fusion of optically-scanned crown and CBCT root into a unique shape.

#### 2.3.1. Spatial Referring Optically-Scanned Tooth Crowns into CBCT Data Sets

**Figure 8.**
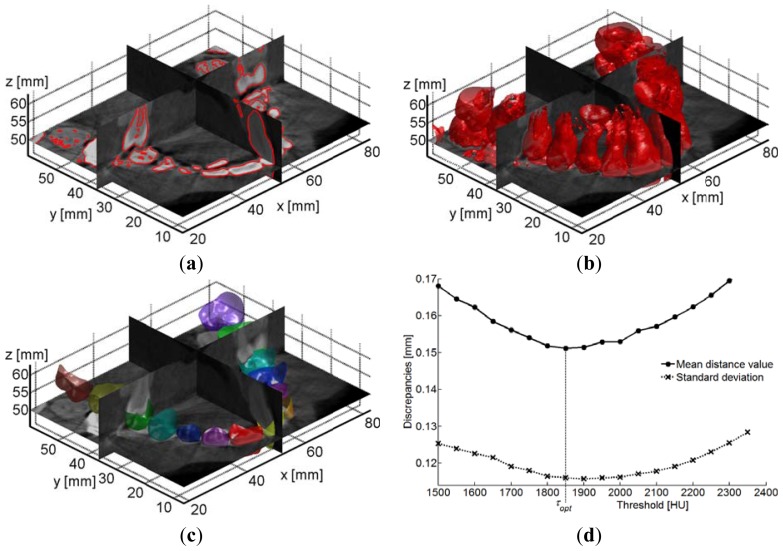
(**a**) Segmentation of CBCT images. (**b**) 3D polygonal mesh from multi-slice processing. (**c**) Overlapping of the optically-scanned crown model to the CBCT data set. (**d**) Mean discrepancies and standard deviations obtained by using different threshold values in the reconstruction of tooth crowns from DICOM images.

The first step involves the reconstruction of crown shapes by segmenting a sequence of Digital Imaging and Communications in Medicine (DICOM) images (slices) obtained from a CBCT scan. Automatic image segmentation of each slice is performed by extracting iso-gray contour lines (Figure 8(a)). An iso-gray surface is then reconstructed from the volume data set (Figure 8(b)). Different models are obtained by varying the threshold gray value. In this paper; an optimal threshold value is determined by using the optically-scanned crown model as ground truth. In particular; the optically-scanned crown model is imported within the DICOM volume data set (Figure 8(c)) and aligned to each different model. The alignment is performed by manually identifying at least three common points and refined by best fitting techniques. Mating surfaces to be used in the refinement of the alignment process are identified within a distance value (*δ_ε_* = 1 mm) in order to avoid misalignments due to noisy data. An optimal threshold (*τ_opt_*) is computed as the value which minimizes the discrepancies between the overlapping crown tissues reconstructed by the optical and CBCT imaging technologies. Figure 8(d) reports the mean discrepancies and the standard deviations obtained by using different threshold values (expressed in Hounsfield Unit scale) to segment DICOM images.

This procedure guarantees an accurate placement of both optically-scanned crown surfaces and soft tissues with respect to the CBCT alveolar bone structure avoiding subjective visual perceptions during the reconstruction process. However, the use of a threshold value provides a unique 3D model of both dentition and bone. Moreover, tooth roots are hardly distinguishable from the surrounding bone. Once the overall optically-scanned model of the tooth crowns has been placed in the correct anatomical position, individual crown tooth shapes are used to guide the extraction of tooth geometries (crown and root) from DICOM volumes.

#### 2.3.2. Reconstruction of Tooth Roots and Multi-Body Modeling

In this paper, an edge-based active contour model has been implemented through a level set formulation. The procedure consists in processing CBCT stack images to retrieve individual tooth contours slice by slice. A closed contour *C* is implicitly represented as the zero level set of a signed distance function *ϕ*, called Level Set Function (LSF), by *C* = {(x,y) | *ϕ*(x,y)=0}. The contour motion, defined as the evolution of the LSF, is given by the zero level set at the time t of the function *ϕ*(*x,y,t*). The basic idea is to allow the zero level set (contour) to deform accordingly to an evolution partial differential equation so as to minimize a given energy functional in order to achieve the target segmentation. The level set method presents properties which are suitable for tooth segmentation of DICOM images [12]. In particular, the implicit representation of the contour avoids the re-parameterization of the curve thus efficiently handling topological changes, such as splitting and merging, in a natural and efficient way (*i.e.*, the tooth contour may split from the single crown into several roots). In the present paper, the Distance Regularized Level Set Evolution (DRLSE) method presented in [13] has been used. DLRSE is a generalized variational level set formulation with a distance regularization term and an external energy term that drives the motion of the zero level contour toward desired locations. Let *I* be an image on a 2D domain Ω, the level set evolution is derived as a gradient flow minimizing a certain energy functional, which is expressed as:

(5) $$\text{E}(\phi )=\mu \int_{\Omega }p\left(\left|\nabla \phi \right|\right)\text{dxdy}+\lambda \int_{\Omega }g\delta \left(\phi \right)\left|\nabla \phi \right|\text{dxdy}+\alpha \int_{\Omega }gH\left(-\phi \right)\text{dxdy}$$

where *μ* > 0, *λ* > 0, *α* is a signed weight coefficient, *δ* is the Dirac function, and *H* is the Heaviside step function. The edge indicator function *g* is defined as:

(6) $$g=\frac{1}{1+{\left|\nabla G_{\sigma }*I\right|}^{2}}$$

where *G_σ_* is a Gaussian kernel with a standard deviation *σ*. The convolution in Equation (6) is used to reduce the noise in the original image. Low values (close to 0) outline the object boundaries, while high values (close to 1) describe homogeneous background. The first term on the right end side of relation Equation (5) acts as the distance regularization term, which is defined with a double-well potential function *p*, and is used to penalize the deviation of *ϕ* from a signed distance during its deformation. The second term makes the contour smooth. The third term is introduced to speed up the motion of the zero level contour during the level set evolution process making the contour either shrink or expand depending on the sign of *α*. The initialization of the LSF is done by using a binary step function, which takes negative values inside the zero level contour and positive values outside. In this case, if the initial contour is placed outside the region to be segmented, the parameter *α* should be positive in order to make the contour shrink in the level set evolution. If the initial contour is placed inside the region, the parameter *α* should take negative values to expand the contour.

**Figure 9.**
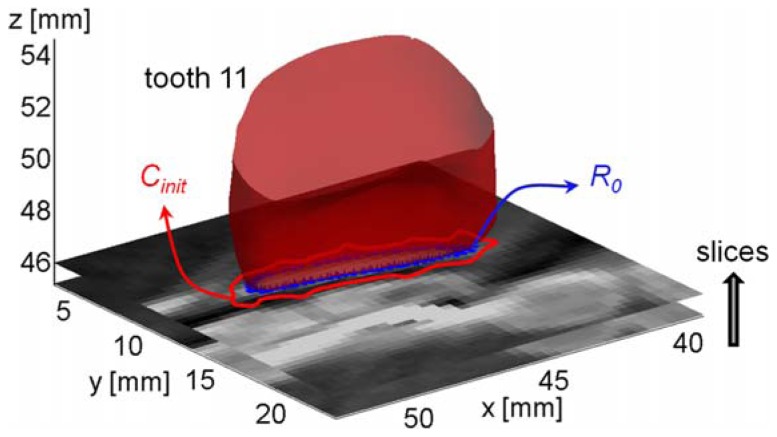
Initialization of the LSF for tooth 11 by using the optically scanned crown.

A fast and accurate convergence of the zero level contour depends on both the involved parameters and the initialization of the closed contour *C*. In [13], a preliminary segmentation step based on a thresholding method is suggested in order to obtain a proper initial binary LSF. In [12], the segmentation of the DICOM images stack is performed by manually drawing the initial contour on a starting slice which is chosen in order to separate the tooth into the crown and root geometries. In this paper, the initialization of the LSF is obtained by exploiting the segmented shapes of optically-scanned crowns, which are spatially referred to the DICOM data sets. The initialization procedure allows the detection of a starting contour, which is close to the tooth region to be segmented. In particular, the procedure can be schematized as follows:

- selection of the individual tooth to be segmented (*T_i_*);
- detection of the preliminary teeth crown region, *R_0_*, by searching the first slice of the whole stack which yields a closed intersection with the aligned optically-scanned tooth *T_i_* (Figure 9);
- definition of the initial contour (*C_init_*) for the level set method by applying a dilation on the detected region *R_0_* using a disk shaped structuring element;
- application of the DRLSE method to the first slice using *C_init_* to initialize the LSF;
- use of the result at initial contour for the DRLSE method on the successive slice.

The proposed methodology provides a straightforward LSF initialization, which is also close to the region to be segmented for each slice. Thus, a few iterations are needed to move the zero level contour from the initial estimate to the desired 2D shape boundary. The parameters of the DRLSE method are experimentally set in order to minimize the discrepancies between the optically-scanned and DICOM crown models. In particular, small positive values of the parameter *α* (from 0 to 1) are used since low shrinking forces have to be applied due to the outer proximity of the initial contour to the tooth shape.

**Figure 10.**
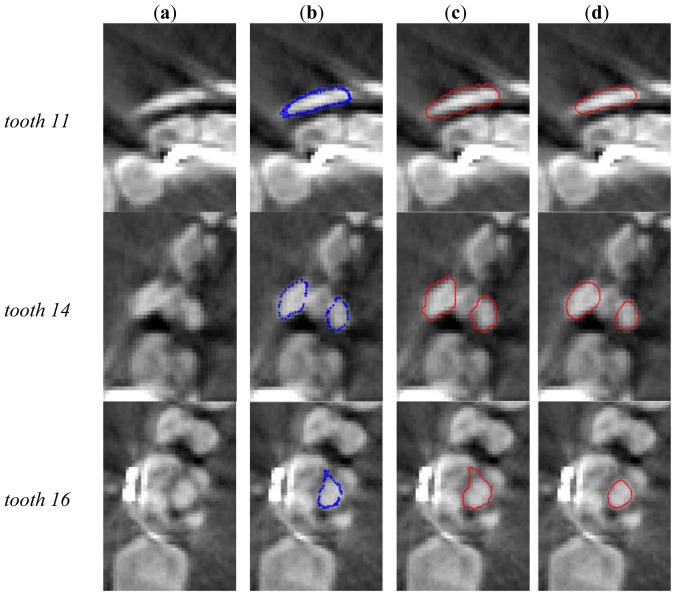
Contour extractions of three teeth by processing the first slices: (**a**) original images; (**b**) contouring initializations; (**c**) initial definitions of contours for the level set method; (**d**) results of the DRLSE method.

Figure 10 show the initializations of the tooth contours applied to three different teeth numbered as tooth 11, 14 and 16 in accordance with the ISO 3950 notation [14]. Figure 10(a) presents the original images while Figure 10(b) illustrates the initial closed contours (blue loops) obtained by slicing the relative optically-scanned crown models aligned to the DICOM stack images. Figure 10(c) shows the dilated contours used as starting binary LSF.

The convergence results of the DRLSE method applied to the same slices are illustrated in Figure 10(d). This procedure automatically restricts the regions of interest for the level set evolution, thus properly constraining the overall process and for the succeeding slices.

**Figure 11.**
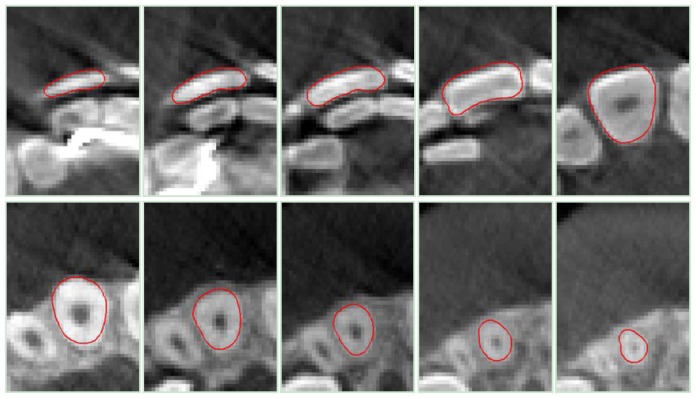
Results of the DRLSE method for *tooth 11*, obtained by processing some CBCT slice images.

**Figure 12.**
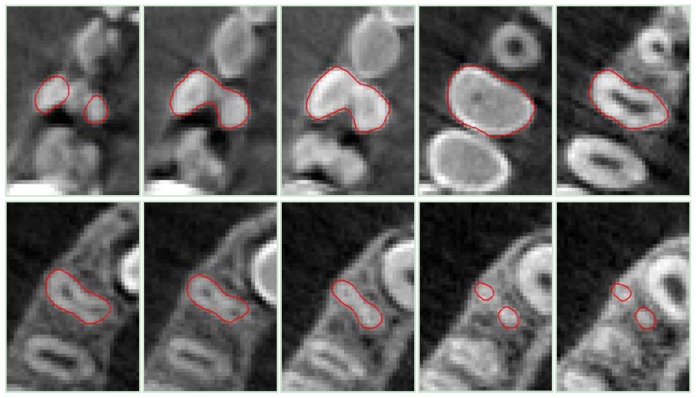
Results of the DRLSE method for *tooth 14*, obtained by processing some CBCT slice images.

**Figure 13.**
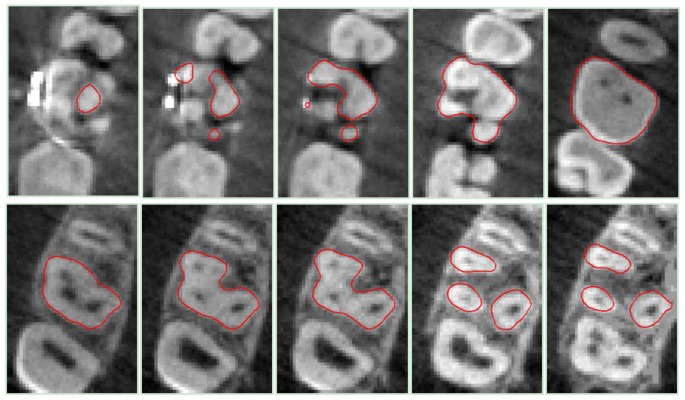
Results of the DRLSE method for tooth 16, obtained by processing some CBCT slice images.

Figures 11, 12 and 13 show the sequential contours obtained by processing the DICOM slices for tooth 11, 14 and 16, respectively.

**Figure 14.**
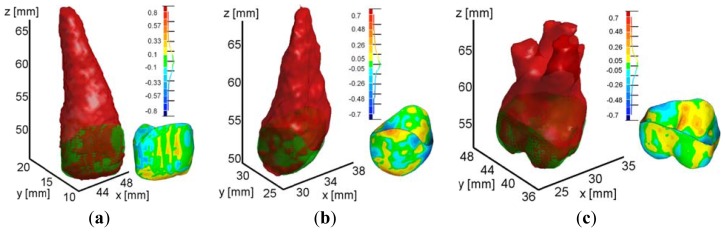
3D CBCT reconstructions with overlapped optically-scanned crowns of *tooth* 11 (**a**), 14 (**b**) and 16 (**c**).

Figure 14 illustrates the final three dimensional models obtained by the DRLSE method. The results evidence the entire individual tooth geometries including roots and crowns. Moreover, the procedure intrinsically provides CBCT reconstructions overlapped to the optically-scanned crowns, which can be used as reference models to assess the segmentation accuracy.

The last step of the 3D modeling process consists in merging the shape reconstructions by keeping the optically-scanned crown geometries and the root CBCT models.

## 3. A Case Study

The proposed methodology has been used to reconstruct an orthodontic model of a 14 years old female patient having two impacted teeth in the upper jaw. An impacted tooth results from a situation in which an unerupted tooth is directed against another tooth or in general in a direction different from normally expected. In the presented case study, the two permanent canine teeth (*teeth* 13 and 23), which should replace the retained primary canines, have not yet erupted in their regular position, even if the normal eruption period is expired. Among the environmental factors that can lead to impacted canines, overcrowding conditions are the most common. These conditions cause a lack of space for the mouth to allow the permanent teeth to move forward and assume their permanent placements. Moreover, impacted teeth can grow at angles toward other teeth, thus forcing them to push against other teeth. In these cases, it is important to treat the impacted teeth in order to prevent improper eruptions or other negative changes in the jaw. The position of the impacted teeth, the disposition of the adjacent teeth as well as the condition of the gingival tissues must be carefully evaluated in order to plan an effective treatment. A combination of surgical tooth exposition together with orthodontic treatment to create the essential space is often required to guide the impacted teeth to their proper placements. An orthodontic virtual model including alveolar bone, soft tissues and teeth would then be a valuable support for a correct overall treatment planning.

**Figure 15.**
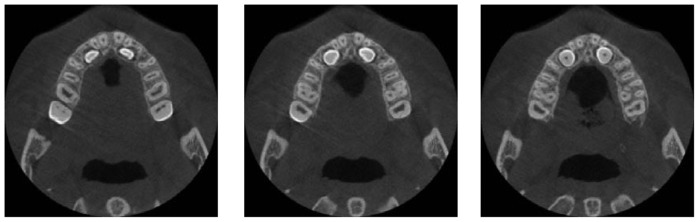
Three slices from the CBCT data set corresponding to the upper jaw.

A patient tomographic scanning has been required in order to fulfill a proper assessment of the clinical conditions. The data set has been generated by the Planmeca^®^ ProMax 3D imaging unit and stored in DICOM files. The resolution of the images is 250 × 250 pixels for a 12 bit depth, while the data set slice thickness is 0.4 mm and pixel spacing 0.4 mm × 0.4 mm. Moreover, the patient's impression has been taken and a plaster dental cast manufactured. An accurate mouth digital model (Figure 4(c)) has then been obtained by integrating the scans acquired by optical scanning both the impression and the plaster cast. The digital patient's mouth model has been finally used to separate the crown tooth region from the gingival tissue (Figure 7). The two distinct data sets obtained from CBCT imaging and surface optical scanning have been integrated as described within section 2.3. Figure 15 show the two impacted canines as visible within three slices from the CBCT data set corresponding to the upper jaw. The canine 3D shapes have been reconstructed using the active contour model. The starting slice as well as the initial contour must be manually selected by the operator since the two impacted teeth are not yet erupted and consequently not visible in the optically-scanned model.

**Figure 16.**
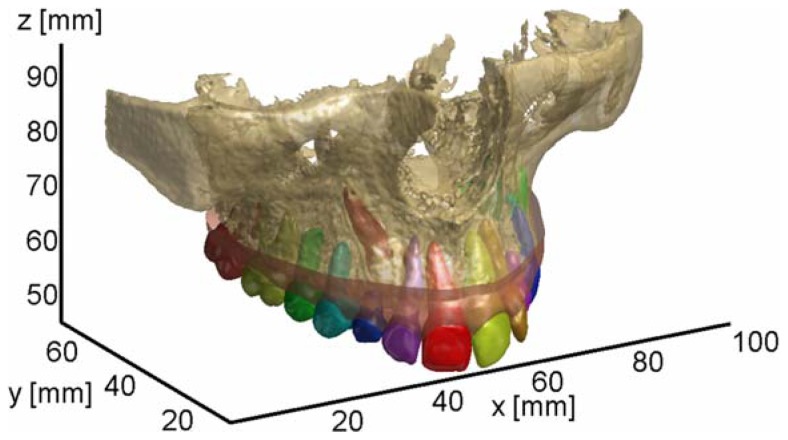
3D views of the multi-body orthodontic model including maxilla, gingival tissue and individual teeth (crowns + roots).

Figure 16 shows the final multi-body orthodontic model including the maxilla, gingival tissue and individual teeth composed of both crown and root regions. The maxilla model has been additionally segmented by following the same approach used for the impacted teeth. Once the initial contour on the starting slice has been manually selected, the level set evolution is performed. For each slice, the areas outlined by the detected teeth contours are then subtracted by the area outlined by the extracted bone contour.

Tooth shapes are then excluded from the alveolar bone model and can be replaced by the separated segmented tooth models. Therefore each tooth can be independently manipulated within the orthodontic model, thus providing an effective tool for orthodontic simulations and treatment planning processes.

## 4. Conclusions

In this paper, a computer-based methodology to digitally reconstruct full multi-body dentitions referred to both gingiva and alveolar bone has been developed. The methodology is based on integrating two imaging sensors, *i.e.*, a structured light scanner and a CBCT device. The optical scanner is used to model tooth crowns and relative gingiva (optically visible surfaces). Moreover, the optically-scanned crown models represent the references for automatic reconstructions of full teeth (including roots) and alveolar bone, through a guided-based processing of CBCT images. The overall methodology intrinsically provides an accurate spatial reference of the optically-scanned dental surfaces within the CBCT volumes. The final model consists of the multi-body data sets, which includes the most accurate model for each tissue: *i.e.*, tooth crowns and gingiva by optical scanning and tooth roots and bone by CBCT imaging.

The proposed approach exploits the guidance of crown tooth shapes obtained by optically scanning patient mouth impressions and plaster casts to robustly perform the complete segmentation of dentition structures from CBCT image sequences. The user does not need to provide anatomical information since the rough initial tooth contour on the first CBCT slice is automatically specified by the aligned optically-scanned tooth models. Therefore, the fusion of 3D orthodontic data is carried out by limiting the requirement of expert human supervision still assuring accurate and consistent identification of the target anatomic structures.

The presented methodology allows the removal of artifacts generated in the reconstruction of tooth shapes owing to the presence of metallic restorations. When metallic dental fillings or orthodontic devices are present, the quality of tomographic scans is greatly reduced (as shown by examples *tooth* 11 and 16 of Figure 10(a)), providing poor reconstructions of 3D shapes in the surroundings of restorations. Crowns surface models obtained by optically scanning impressions and plaster casts are used to remove the corrupted information from DICOM data sets. Moreover, accuracy and resolution of the optical scanner as compared to a CBCT scanning allows optimal reconstructions of both tooth crown surfaces and oral soft tissues making feasible the design and production of tight-fitting removable appliances to be used for orthodontic treatments.
